# Centering Veteran expertise: Advancing Peer-Led Harm Reduction in the Veterans Health Administration

**DOI:** 10.1186/s12954-026-01478-y

**Published:** 2026-08-01

**Authors:** Beth Dinges, Tessa Rife-Pennington, Avery Risinger, Aaron Ferguson, Aaron M. Laxton, Kristi Rodriguez, Zachary Goff, Franklin Taveras, Juan Cortez, Alex S. Bennett, Nikketa Marina Burges, Mark Jenkins, Garry Hayes, Minh Q. Ho

**Affiliations:** 1 VA Illiana Healthcare System, 1900 E Main St, Danville, IL 61832 USA; 2 San Francisco Veterans Affairs Health Care System, 4150 Clement St (119), San Francisco, CA 94121 USA; 3 School of Pharmacy, University of California, San Francisco, UCSF, 513 Parnassus Ave, Box 0403, San Francisco, CA 94143 USA; 4 Community Medical Services, 1101 Arrow Point Dr. Suite 214, Cedar Park, TX 78613 USA; 5 Saint Louis University, 1 N. Grand Blvd, Saint Louis, MO 63103 USA; 6 Community Medical Services, Southern Arizona, 3720 S. Park Ave Suite 601, Tucson, AZ 85713 USA; 7 Montare Behavioral Health/Renewal Health Group, 829860 Copper Run Dr, Harvest, AL 35749 USA; 8 NYU School of Global Public Health, 708 Broadway 6th Floor, Rm 658A, New York, NY 10003 USA; 9 Department of Anthropology10, University of Colorado Denver, Campus Box 103, PO Box 173364, Denver, CO 80217 USA; 10 Connecticut Harm Reduction Alliance, 28 Grand St, Hartford, CT 06106 USA; 11 Independent harm reduction advocate, Chicago, IL USA; 12 VA Orlando Healthcare System12, 13800 Veterans Way, Orlando, FL 32827 USA

**Keywords:** Veterans, Lived expertise, Harm reduction, Peer support, Substance use, Veterans health, Social justice

## Abstract

The principle “Nothing about us without us” underscores the ethical imperative to involve people with lived and living expertise in shaping harm reduction praxis. While the United States Veterans Health Administration (VHA) has expanded harm reduction initiatives, such as syringe services and naloxone distribution, over the past four years, Veterans with lived and living expertise (VWLLE) have not been meaningfully integrated into policy and program design. In response, VHA clinicians and advocates established the Harm Reduction Veteran Engagement Board (HR-VEB), a peer-supported initiative that positions VWLLE as partners in harm-reduction reforms. This perspective emphasizes structural barriers faced by Veterans who use drugs, highlights the leadership and work of HR-VEB members, and outlines policy and practice recommendations for designing more equitable care. Through the voices and work of Veterans, we argue that peer-led harm reduction is both evidence-based and a matter of social justice.

## Background: harm reduction, social justice, and Veteran-led models

Harm reduction is a pragmatic, public health, and human rights approach to minimizing the harm associated with drug use without requiring abstinence. Originating in the late 20th century in response to the HIV/AIDS epidemic, harm reduction strategies such as syringe services programs (SSPs), overdose education and naloxone distribution, and HIV preexposure prophylaxis have shown efficacy and are currently global standards of care [1–4].

As the most extensive integrated healthcare system in the United States, the U.S. Department of Veterans Affairs (VA) provides care for approximately 9 million Veterans annually. Within VA, the Veterans Health Administration (VHA) cares for a population with higher rates of substance use, yet harm reduction strategies remain underutilized [5]. While the VHA has made significant progress through expansive naloxone distribution [6] and the establishment of SSPs at more than 40 medical centers, there remain opportunities to further enhance these programs through the flexibility and cultural competence of community-based and peer-led models.

The Harm Reduction Veteran Engagement Board (HR-VEB), founded in 2024 by VHA clinicians, Veterans who use drugs, and Veteran advocates, operates as a collaborative epistemic space where Veterans and clinicians together co-produce harm reduction knowledge, challenging traditional hierarchies of expertise within the VHA.

HR-VEB resembles Community advisory boards (CABs) which are widely used to ensure community voices are included in decision-making across public health and policy settings [7]. HR-VEB has a distinctive focus on centering VWLLE in shaping harm reduction strategies. Unlike traditional recovery-focused VA boards, HR-VEB is designed to inform structural change through meaningful peer engagement and advocacy.

This perspective has three aims: to emphasize structural barriers faced by Veterans who use drugs, highlight the leadership work of HR-VEB members, and outline policy and practice recommendations for designing more equitable care.

## Structural barriers to care for Veterans

Substance use among Veterans is shaped by trauma–before, during, and after service. Veterans have more adverse childhood experiences [8], compounded by military-specific trauma such as combat, military sexual trauma, traumatic brain injury, and chronic pain [9, 10]. Veterans with “Other Than Honorable” (OTH) discharges are subject to particularly intense stigma, both inside and outside healthcare systems. They are often excluded from VA healthcare benefits, creating a “missing middle” population with few safety nets [11, 12, 13]. OTH discharges represent approximately 500,000 Veterans alive today; the rate of this type of discharge has increased since the post-9/11 era [9, 14]. Additionally, there is an overrepresentation of Black and Latinx Veterans and survivors of military sexual trauma [15, 16]. OTH discharges are associated with disproportionately high rates of homelessness, incarceration, suicide, and overdose [17, 18]. VHA data on suicides and overdoses largely exclude Veterans with OTH discharges, limiting their visibility and the urgency of policy change [19].

This intersection of trauma and systemic neglect is exemplified by one HR-VEB member, a former US Navy Veteran: “After years of homelessness and multiple overdoses, I know firsthand how institutional barriers and stigma block access to life-saving care. Being discharged for marijuana use denied me benefits, but it did not stop me from becoming a harm reduction advocate.”

### Gaps in Va harm reduction implementation

Due to misinterpretations of federal funding restrictions, VHA facilities did not begin purchasing syringes for distribution until 2021, well into the opioid crisis. Clarification from the Network for Public Health Law’s Harm Reduction Legal Project paved the way for a VHA memo explicitly permitting syringe purchases [20, 21]. Even afterward, institutional barriers such as patient-specific prescription requirements and electronic health record documentation for syringes and naloxone discourage participation and conflict with harm reduction best practices [22–24].

People with lived and living expertise possess unique cultural capital to build trust and deliver resources in settings inaccessible to traditional providers. Yet they are often excluded from program planning, underpaid, undervalued, and face job insecurity and burnout [25, 26]. Within VHA, this exclusion is acute: peer specialists, Veterans trained to support mental health recovery [27], typically work under abstinence-only models that exclude active drug users, reinforcing stigma and undermining evidence-based peer-delivered harm reduction [28, 29].

Veterans cite stigma, trauma, and prior negative healthcare encounters as reasons for disengaging from VHA care, often emphasizing the need for more VWLLE staff to build trust and cultural familiarity [33]. Peer-delivered syringe access and naloxone distribution are evidence-based practices but remain prohibited by current VHA policies, a key missed opportunity [28].

## Centering veteran expertise: harm reduction Veteran engagement board

### Formation and membership

Recognizing these gaps, VHA clinicians from Danville, Illinois; Orlando, Florida; and San Francisco, California, who began piloting access to sterile syringes and drug-checking strips within VHA in 2017, collaborated with community partners to establish the HR-VEB. While not a formal research project, the Board embodies principles of community‑based participatory research. Veterans were recruited through harm reduction networks, VHA programs, and word‑of‑mouth referrals from existing members, with intentional efforts to include Veterans of color, women and gender‑diverse Veterans, lesbian, gay, bisexual, transgender, queer or questioning, intersex, asexual, and more (LGBTQ+) Veterans, those with OTH discharges, and VWLLE of substance use, homelessness, incarceration, and HIV and viral hepatitis.

## Operating processes

Meeting monthly since March 2024, HR‑VEB has focused on topics such as stigma, treatment barriers, peer roles, and VA-community collaboration. Agendas are co‑developed by Veteran members and clinician co‑facilitators, and meetings are guided by ground rules emphasizing respect, confidentiality, non‑punitive discussion of use, and non‑abstinence expectations. Decisions are made through a modified consensus process, with draft documents and core materials circulated in advance to support informed discussion. Guest presenters are invited to share materials and receive structured consultative input from members. Despite these strengths, co‑facilitators have thus far been unable to overcome institutional barriers to providing financial compensation to members, highlighting an important area for future system change.

## Activities and leadership

Members’ presentation on a national VA program office call marked the first time that VWLLE spoke about harm reduction within the VHA. Members have informed newer VA harm reduction programs, co-developed safer use educational materials, and participated in national VA staff training. They’ve presented at Veteran Service Organizations, academic forums, and national conferences, fostering collaboration between VHA and community organizations [34–36]. And their advocacy represents a significant shift in peer-led policy influence within VHA, although broader adoption and formal evaluation remain necessary to assess scalability.

Moreover, HR-VEB members are leaders in community-based harm reduction. Two work as outreach workers and peer advocates within New York University’s National Institutes of Health (NIH)-funded Project Respond, connecting unstably housed Veterans to harm reduction supplies, education, and resources [37]. One HR-VEB member and founder of a statewide harm reduction organization donated its mobile units stocked with harm reduction supplies to VHA facilities, helping circumvent internal procurement and access barriers. Other members connect Veterans with harm reduction supplies and access to lifesaving medications for opioid use disorder, lead national peer advocacy organizations and harm-reduction-focused podcasts, coordinate referrals through the VA Community Care Program, develop state-funded mobile treatment units, and conduct graduate-level research in medical anthropology and addiction. Collectively, they leverage their lived and living experience in multiple roles as outreach workers, organizational leaders, advocates, researchers, and peer supporters, to expand harm reduction access for Veterans both inside and outside the VA.

These initiatives illustrate the power of peer-led harm reduction and the potential to supplement institutional gaps in healthcare-based delivery. Implementing meaningful change requires systematic policy reform guided by evidence and community expertise. The following recommendations outline concrete steps for embedding peer leadership into VHA harm reduction systems while advancing broader social justice goals.

### HR‑VEB and research

HR-VEB was founded by three VHA clinicians pioneering syringe and drug-checking strip access at VHA facilities not as a research initiative, but to address critical gaps in harm reduction efforts, particularly the absence of meaningful inclusion of people with lived or living experience. To date, the Board has declined invitations for one‑time or symbolic consultation, prioritizing deeper collaborative roles. Consistent with community-based participatory research, future priorities include longer-term research partnerships in which VWLLE are co-investigators, co-authors, and co-designers of implementation strategies. Ultimately, HR‑VEB aims to function as a standing community structure supporting VHA‑based harm reduction implementation and research, including work focused on Veterans with other than honorable discharges and other marginalized groups.

## Policy and practice recommendations

The following recommendations emerged through collaborative discussions among HR-VEB members and VA clinicians between March 2024 and August 2025. Reflecting the Board’s commitment to shared authority and to recognizing lived and living experience as vital epistemological expertise within institutional decision-making, these recommendations were developed in response to challenges specific to VA. These recommendations offer a transferable framework for integrating peer-led harm reduction approaches across healthcare and broader public health systems. Translating these changes beyond VA could amplify the impact of VWLLE and extend the reach of evidence-based harm reduction interventions.

## Improve support for Veterans with OTH discharges

Structural problems require systemic solutions, not punitive responses to individual survival strategies developed during military service. While recent policy changes have improved crisis access for Veterans with OTH discharges, full VA benefits remain tied to a lengthy, often unsuccessful appeal process [9]. Ideally, a more just and therapeutic approach should begin within the military itself by treating substance use as a health issue rather than a disciplinary infraction. At minimum, stronger tracking of outcomes for Veterans with OTH discharges through cross-agency coordination between the Department of Defense and VHA would increase visibility and better inform policy [9].

### Strengthen community partnerships

Legal guidance from the Network for Public Health Law’s Harm Reduction Legal Project helped correct widely held, but inaccurate, interpretations of federal funding restrictions [38]. Expanded collaborations with community-based organizations (CBOs) and public health departments can help remove remaining barriers, expand access to harm reduction, and strengthen safety nets, especially for Veterans excluded from VHA care.

### **Designate harm reduction funding for community initiatives**

The VA’s Staff Sergeant Fox Suicide Prevention Grant Program funds mental health and suicide prevention while promoting harm reduction [39]. Research supports expanding inclusion criteria or creating parallel funding mechanisms; a similar grant program focused on harm reduction could support peer-led services and expand outreach beyond the VA’s standard services.

### Integrate overdose prevention with suicide prevention

The nexus between suicide, trauma, and substance use requires an approach that bridges community and healthcare [40]. Operation Deep Dive investigates social and structural determinants of health behind Veteran suicides, including overdose deaths with unidentified intent. Their findings suggest that many “accidental” deaths reflect cumulative structural failures, including loss of purpose, housing instability, criminalization, and lack of care [41]. Importantly, the Veteran co-led study includes data on Veterans with OTH discharges. Ensuring coordination between programs for suicide prevention and harm reduction–by including harm reduction as a component in suicide prevention efforts and vice versa–could allow for more holistic interventions.

### Revisit policies for peer specialist roles

Authorizing Peer Specialists and other outreach workers to distribute harm-reduction supplies (e.g., syringes, naloxone, drug-checking strips) may improve access. Furthermore, expanding peer-delivered services to include point-of-care testing (e.g., HIV, STI, and HCV testing) should be explored, with appropriate training, funding, and fair compensation [42, 43]. Broadening VA pathways for employing VWLLE, such as through “excepted service” hiring authorities or VA-administered grants to community-based organizations, would foster a more inclusive and responsive workplace.

### Reduce prescription- and documentation-related barriers

Institutions have successfully implemented SSPs without charting requirements (e.g., University of Miami’s Infectious Disease Elimination Act Exchange) [44]. Reviewing current policies on naloxone and syringe distribution, particularly regarding electronic health record charting and prescribing rules, could help identify opportunities to reduce stigma and improve access.

### Support Veteran engagement boards

Created to ensure transparency, the Federal Advisory Committee Act can hinder meaningful involvement of affected Veterans [45]. Exploring alternative structures for peer engagement, especially those that allow for compensation and co-leadership, could ensure sustained participation while maintaining accountability and transparency.

## Conclusions

HR-VEB signifies a meaningful shift from top-down systems toward peer-driven, justice-centered care. In recognizing lived/living expertise, embedding harm reduction into institutional policy, and confronting structural inequalities, the VHA has the potential to become a leader in equitable, evidence-based care. Harm reduction is not only simply clinically effective—it is our moral and public health imperative.

The HR-VEB model extends the principle of *“Nothing about us without us”* beyond rhetoric; it demonstrates how centering lived and living expertise transforms institutional care. Its broader applicability lies in showing that the same peer-led frameworks capable of reforming the VHA might also work to repair the harms of the “wars on drugs” that have, for decades, decimated our warfighters, eroding the trust between Veterans and the systems intended to serve them.

## Data Availability

No datasets were generated or analysed during the current study.

## Abbreviations

VA: U.S. Department of Veterans affairs
VHA: Veterans hHealth administration
OTH: Other than honorable
SSPs: Syringe services programs
HR-VEB: Veteran engagement board
VWLLE: Veterans with lived and living expertise

## Declarations

### Authors’ information

HR-VEB facilitators and collaborators include Veterans with lived and living expertise, clinicians from VHA harm reduction programs, community harm reduction leaders, and academic researchers from universities and CBOs.
